# Psychometry as a Means of Diagnosis

**Published:** 1888-02

**Authors:** 


### PSYCHOMETRY AS A MEANS OF DIAGNOSIS.

### IMPORTANT TO THE SUFFERING.

The wonderful gift of determining the nature and condition of things by being brought into contact with tangible elements which serve as a connecting link between the present and the past, the living and the so-called dead, or the living subject and the sensitive has often been turned to profitable account as a valuable adjunct to the physician in his efforts to arrive at the real seat of disease, and point unerringly to the surest means of relief.

For several months past we have in various instances tested the accuracy of psychological diagnosis of diseases through the instrumentality of a delicately organized sensitive, who is also a regular graduated physician of an established and well reputed school of medicine, for many years largely and successfully engaged in the practice of his profession in New York City, and we have in this manner satisfied ourself of the great value to the afflicted, of this subtle and mysterious agent, when called into exercise by one qualified to follow out its teachings.

Acting upon the knowledge we have thus acquired by close observation, it gives us pleasure to state that we have been able to secure the services of the Sensitive in question, in connection with Hall's Journal of Health, so that such of our readers as are afflicted by disease, may through this open door obtain a psychological reading of their ailments upon forwarding to this office anything that will establish sympathetic relations with the sensitive; as for example, a letter or fragment of writing, or a lock of hair. See advertisement of Dumont C. Dake, M.D.

---

An itemized bill of the twelfth century is old enough, in all conscience, but this one from the records of Winchester Cathedral, dated 1182, may be new to most people :

| | S. | D. |
|---|---|---|
| For work done in soldering and repairing St. Joseph.................... | 0 | 8. |
| Cleaning and ornamenting the Holy Ghost............ .. ............ | 0 | 6. |
| Repairing the Virgin Mary before and behind, and making a new child.. | 4 | 8. |
| Screwing a nose in the Devil, putting in the hair in his head and placing a new joint in his tail........................................ | 5 | 6. |

The total bill amounted to 11 shillings and 4 pence, and it is to be presumed that the workman got his pay, though the records are silent on the point.

